# Effects of decreased lactate accumulation after dichloroacetate administration on exercise training–induced mitochondrial adaptations in mouse skeletal muscle

**DOI:** 10.14814/phy2.12555

**Published:** 2015-09-28

**Authors:** Daisuke Hoshino, Yuki Tamura, Hiroyuki Masuda, Yutaka Matsunaga, Hideo Hatta

**Affiliations:** 1 Department of Sports Sciences, The University of TokyoTokyo, Japan

**Keywords:** Dichloroacetate, exercise training, lactate, mitochondria, skeletal muscle

## Abstract

Recent studies suggested that lactate accumulation can be a signal for mitochondrial biogenesis in skeletal muscle. We investigated whether reductions in lactate concentrations in response to dichloroacetate (DCA), an activator of pyruvate dehydrogenase, attenuate mitochondrial adaptations after exercise training in mice. We first confirmed that DCA administration (200 mg/kg BW by i.p. injection) 10 min before exercise decreased muscle and blood lactate concentrations after high-intensity interval exercise (10 bouts of 1 min treadmill running at 40 m/min with a 1 min rest). At the same time, exercise-induced signal cascades did not change by pre-exercise DCA administration. These results suggested that DCA administration affected only lactate concentrations after exercise. We next examined the effects of acute DCA administration on mRNA expressions involved with mitochondrial biogenesis after same high-intensity interval exercise and the effects of chronic DCA administration on mitochondrial adaptations after high-intensity interval training (increasing intensity from 38 to 43 m/min by the end of training period). Acute DCA administration did not change most of the exercise-induced mRNA upregulation. These data suggest that lactate reductions by DCA administration did not affect transcriptional activation after high-intensity interval exercise. However, chronic DCA administration attenuated, in part, mitochondrial adaptations such as training-induced increasing rates of citrate synthase (*P *=* *0.06), *β*-hydroxyacyl CoA dehydrogenase activity (*P *<* *0.05), cytochrome c oxidase IV (*P *<* *0.05) and a fatty acid transporter, fatty acid translocase/CD36 (*P *<* *0.05), proteins after exercise training. These results suggest that lactate accumulation during high-intensity interval exercise may be associated with mitochondrial adaptations after chronic exercise training.

## Introduction

Lactate is produced and accumulated in muscle and blood during exercise, especially high-intensity exercise. We currently understand that lactate is a useful energy fuel but not a cause of fatigue (Robergs et al. 2004; Kitaoka et al. 2012). Recently, lactate has been considered a signal molecule that induces various adaptations in several tissues, such as specific gene expression (Latham et al. 2012), mitochondrial biogenesis (Hashimoto et al. 2007), angiogenesis (Porporato et al. 2012), and lipolysis (Hashimoto et al. 2013). One of the main physiological adaptations induced by lactate is mitochondrial biogenesis in skeletal muscle cells. Hashimoto et al. reported that lactate stimulation increased levels of peroxisome proliferator–activated receptor gamma coactivator 1-alpha (PGC-1alpha), which is a master regulator of mitochondrial biogenesis, and mitochondrial protein expression in L6 cells (Hashimoto et al. 2007). In addition, PGC-1alpha mRNA expression increased after exercise above the intensity of lactate threshold (LT), but not below the LT in humans (Tobina et al. 2011). These results raised the hypothesis that lactate accumulation during exercise is associated with exercise-induced mitochondrial biogenesis in vivo. However, whether lactate accumulation is required for the exercise-induced mitochondrial biogenesis in vivo is unknown.

High-intensity exercise increases lactate production in working muscles because of the acceleration of glycogenolysis and glycolysis. Lactate is converted to pyruvate in the cytosol, and then oxidation is started via the reaction of mitochondrial pyruvate dehydrogenase (PDH), which converts pyruvate into acetyl-CoA in mitochondria. Enhanced PDH activation decreases lactate production and enhances lactate clearance because the glycogenolytic flux is greater than the PDH flux during even submaximal exercise (Howlett et al. 1998). Dichloroacetate (DCA) is known as a PDH activator. Previous studies reported that the infusion or injection of DCA before exercise decreased muscle and blood lactate concentrations during and after exercise in rodents (Schneider et al. 1981; Hatta et al. 1991; Durkot et al. 1995). It is possible that different exercise intensities result in different lactate accumulations during exercise, but signal cascades involved with mitochondrial biogenesis are increased by exercise intensity dependent manner (Chen et al. 2003; Egan et al. 2010). In addition, DCA is effective in the PDH activation when exercise is of high intensity (submaximal) and short duration (Howlett et al. 1999; Parolin et al. 2000; Savasi et al. 2002). Therefore, the combination of high-intensity interval exercise and DCA administration makes it possible to examine whether differences in lactate accumulation during exercise of the same intensity can affect exercise-induced mitochondrial biogenesis in skeletal muscle.

Therefore, we hypothesized that reduced lactate accumulation following DCA administration attenuates muscle mitochondrial biogenesis after exercise training. We first confirmed pre-exercise DCA administration by i.p. injection decreased muscle and blood lactate concentrations immediately after high-intensity exercise. We next examined whether acute pre-exercise DCA administration inhibits increases in mRNA expressions involved with mitochondrial biogenesis after acute exercise and whether chronic pre-exercise DCA administration attenuates mitochondrial adaptations such as increases in mitochondrial enzyme protein content and/or activity after exercise training.

## Materials and Methods

### Animals

ICR male mice (6 weeks of age; CLEA Japan, Tokyo) were used in this study. The mice were housed on a 12:12-h light–dark cycle in an air-conditioned room. The animals were provided with standard chow and water ad libitum. All experimental treatments were approved by the University of Tokyo committee on animal care.

### Experimental design

#### Experiment 1: effects of pre-exercise DCA administration on metabolites (lactate and glycogen) and signal cascades after high-intensity interval exercise

ICR male mice were divided into saline (Saline, *n *=* *6) and DCA (*n *=* *6) group. Mice performed 10 bouts of 1 min of treadmill running at 40 m/min with 1 min of rest. DCA and saline groups were injected with DCA (200 mg/kg) or the same volume of saline, respectively, 10 min before treadmill running. This DCA injection method followed a previous study reporting decreases in muscle and blood lactate concentrations (Hatta et al. 1991). The blood was obtained from their tails to measure lactate concentration at basal state. The gastrocnemius muscle and blood were harvested immediately after exercise to analyze metabolites (lactate and glycogen) and signal cascades.

#### Experiment 2: effects of acute and chronic DCA administration on mitochondrial adaptations after high-intensity interval exercise

##### Acute experiment

ICR male mice were divided into the saline-sedentary (*n *=* *6), the DCA-sedentary (*n *=* *6), the saline-exercised (*n *=* *6), and the DCA-exercised (*n *=* *6) groups. Gastrocnemius muscles in the DCA- and saline-sedentary groups were harvested 10 min after the injection of DCA (200 mg/kg) or the same volume of saline. The saline- and DCA-exercised groups performed 10 bouts of 1 min of treadmill running at 40 m/min with 1 min of rest. Gastrocnemius muscles in the exercised mice were harvested 3 h after exercise. The tissues were rapidly frozen in liquid nitrogen and stored at –80°C until the mRNA quantification.

##### Chronic experiment

ICR male mice were assigned to the saline administration without any training (SAL, *n *=* *7), DCA administration without any training (DCA, *n *=* *7), saline administration with training (SAL + T, *n *=* *7), or DCA administration with training (DCA + T, *n *=* *8) groups. The SAL + T and DCA + T groups performed treadmill running training (10 bouts × 1 min of 38–43 m/min running with 1 min of rest) for 4 weeks (3 days/week in week 1 and 5 days/week in weeks 2–4). The intensity was gradually increased over the experimental period (Table 1). The SAL + T and DCA + T groups were administered saline (same amount as DCA) and DCA (200 mg/g BW), respectively, via i.p. injection 10 min before every training session. The SAL and DCA groups were administered the same volume of saline or DCA via i.p. injection on the same days. Gastrocnemius muscles were harvested 72 h after the last session, rapidly frozen in liquid nitrogen, and stored at –80°C until further analysis. We used white region of muscle for analysis because we expected that the muscle would be more mobilized during high-intensity exercise and more changeable after high-intensity exercise training.

**Table 1:** Summary of intensity and total distance in training per week during the experimental period

Training weeks	1	2	3	4
No. of interval/training session	10	10	10	10
No. of training sessions per week	3	5	5	5
Mean speed (m/min)	38	39	41	43
Minimal speed during training session (m/min)	30	38	40	41
Maximal speed during training session (m/min)	40	40	42	45
Mean total running distance/week (m)	1137	1950	2053	2168

### Lactate and glycogen concentrations

Blood lactate concentrations were measured using blood from the opened chest with a lactate meter (Lactate Pro, Arkray, Kyoto, Japan). To measure muscle lactate concentrations, 15 mg of white gastrocnemius muscle tissue were homogenized with 0.6 N HClO_4_ and centrifuged at 3000 *g* for 5 min. Hydrazine/glycine buffer (0.4 mol/L hydrazine, 0.5 mol/L glycine; pH 9.0) was combined with 40 mmol/L *β*-NAD and 0.03 mL of lactate dehydrogenate (5 mg protein/mL) (Gutmann and Wahlefeld 1974). Muscle glycogen content was measured using the phenol–sulfuric acid method (Lo et al. 1970). Briefly, 300 *μ*L of 30% KOH saturated with Na_2_SO_4_ were added to the muscle samples to completely dissolve the muscle. The homogeneous solutions were mixed with 360 *μ*L of 95% ethanol and placed on ice for 30 min. The solutions were centrifuged at 840 *g* for 30 min, and then the supernatant was removed, after which the pellet (glycogen precipitates) was resuspended in 600 *μ*L of distilled water. In total, 200 *μ*L of the sample, 100 *μ*L of phenol, and 96–98% sulfuric acid were combined and incubated for 10 min, and the absorbance was read on a spectrometer at 490 nm.

### Mitochondrial enzyme activities

The activities of citrate synthase (CS) and *β*-hydroxyacyl CoA dehydrogenase (*β*-HAD) were determined in white gastrocnemius muscle homogenates. Specifically, a portion of muscle (6–10 mg) was homogenized in 100 vol/wt of 100 mmol/L potassium phosphate buffer. Citrate synthase and *β*-HAD activities were measured spectrophotometrically using the methods of Srere (1969) and Bergmeyer (1974), respectively.

### Protein preparation and western blot

White gastrocnemius muscle samples were homogenized as described previously (Hoshino et al. 2013; Tamura et al. 2015) using lysis buffer (1% Triton X-100, 50 mmol/L Tris-HCl, 1 mmol/L EDTA, 1 mmol/L EGTA, 50 mmol/L sodium fluoride, 10 mmol/L sodium beta-glycerol phosphate, 5 mmol/L sodium pyrophosphate, 2 mmol/L dithiothreitol, pH 7.5) containing 10 *μ*g/mL each pepstatin A, aprotinin, and leupeptin, 1 mmol/L Na orthovanadate, and 0.177 mg/mL phenylmethylsulfonyl fluoride. The concentrations of sample proteins were measured by the Bradford method. Whole muscle proteins were separated using standard SDS-PAGE procedures (7.5–12% polyacrylamide gels) and transferred to a polyvinylidene difluoride membrane. Membranes were blocked with 7.5% bovine serum albumin for 1 h and incubated overnight with antibodies specific for PGC-1alpha (Calbiochem, La Jolla, CA), cytochrome c oxidase IV (COXIV, Invitrogen, Burlington, ON, Canada), fatty acid translocase/CD36 (FAT/CD36, Abcam, Cambridge, MA), phosphorylated (p)-AMP-activated protein kinase (P-AMPK, Thr172; Cell Signaling Technology [CST] Japan, Tokyo, Japan), AMPK (CST Japan), p-acetyl-CoA carboxylase (P-ACC, Ser79; CST Japan), ACC (CST Japan), p-p38 mitogen-activated protein kinase (P-p38, Thr170/Tyr182; CST Japan), p38 MAPK (CST Japan), p-Ca^2+^/calmodulin-dependent kinase II (P-CaMKII, Thr286; CST Japan), or CaMKII (BD Biosciences Japan, Tokyo, Japan). The membranes were then incubated for 1 h at room temperature with secondary antibodies. Blots were quantified using a scanner (Chemi doc, Bio Rad, Hercules, CA) with appropriate software (Quantity one, Bio Rad). For western blots, equal protein quantities were loaded and verified by GAPDH (Abcam) protein content.

### Quantitative real-time PCR

Total RNA was isolated from white gastrocnemius muscle tissue using Isogen (Nippon Gene, Tokyo, Japan) and digested with DNase 1 (RQ 1, Promega, WI). Reverse transcription was conducted using the PrimeScript RT reagent kit (Takara, Tokyo, Japan) according to the manufacturer’s instructions. cDNA was amplified using Sybr Pre-mix EX Taq II (Takara). Real-time PCR was performed using the Thermal Cycler Dice Real Time System (Takara). Melt curve analysis was performed to ensure that a single PCR product was amplified. All experiments were performed in duplicate. Data quantification was performed with the Thermal Cycler Dice Real Time System software using the 2^–ΔΔ^ method. 18S ribosomal RNA was used as an endogenous control. The sequences of the primers synthesized by Takara are described in Table 2.

**Table 2:** Mouse-specific primer pairs used for quantitative RT-PCR

	Forward	Reverse
RS18	5′-TTCTGGCCAACGGTCTAGACAAC-3′	5′-CCAGTGGTCTTGGTGTGCTGA-3′
CS	5′-CAAGTCATCTACGCCAGGGACA-3′	5′-CAAAGCGTCTCCAGCTAACCAAG-3′
*β*-HAD	5′-AGCACAGACCTGGTGGTGGA-3′	5′-AGACGTGTTGCTGGCAAAGATG-3′
PGC-1alpha	5′-TTGACAGCTGCATTCATTTATCACC-3′	5′-AACACTTGAGCAAGCATTCGACA-3′
COXIV	5′-GAGCTTCGCCGAGATGAACAG-3′	5′-ACCCAGTCACGATCGAAAGTATGAG-3′
FAT/CD36	5′-TTGGCTTGCAACTGTCAGCAC-3′	5′-TGCTGTAGCCAAGAACTCCAGAGA-3′

RS18, ribosomal protein S18; CS, citrate synthase; HAD, hydroxyacyl-coenzyme A dehydrogenase; PGC-1alpha, peroxisome proliferative activated receptor gamma coactivator 1 alpha; COXIV, cytochrome c oxidase subunit IV; FAT/CD36, fatty acid translocase/CD36.

### Statistics

**Figure 1:**
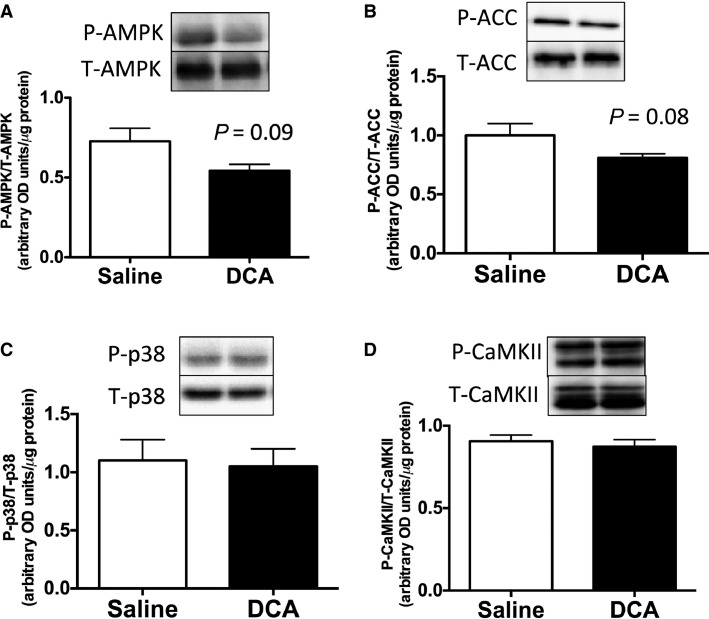
(A) P-AMPK/T-AMPK, (B) P-ACC/T-ACC, (C) P-p38/T-p38, and (D) P-CaMK/T-CaMK expressions in the white gastrocnemius muscle in saline- or DCA-injected mice immediately after high-intensity interval exercise. Values are means ± SE (*N *=* *6). *P*-value versus the saline group.

**Figure 2:**
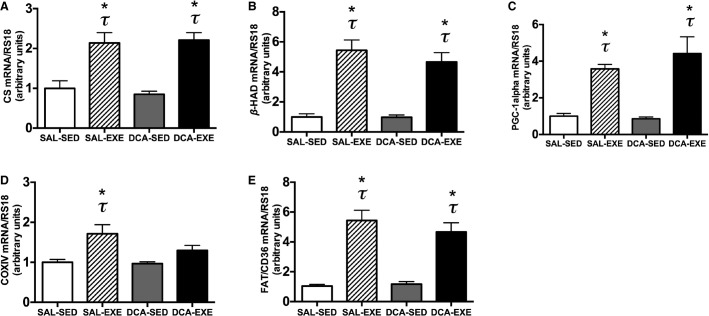
mRNA expression of (A) CS, (B) *β*-HAD, (C) PGC-1alpha, (D) COXIV, and (E) FAT/CD36 in the white gastrocnemius muscle before exercise (sedentary) with 10 min after saline (saline-sedentary group, SAL-SED) or DCA (DCA-sedentary group, DCA-SED) administration and 3 h after high-intensity interval exercise with saline (saline-exercised group, SAL-EXE) or DCA (DCA-exercised group, DCA-EXE) administration. Values are means ± SE (*N *=* *6). *P <* *0.05 versus the SAL-SED group. *τ P *<* *0.05 versus the DCA-SED group.

**Figure 3:**
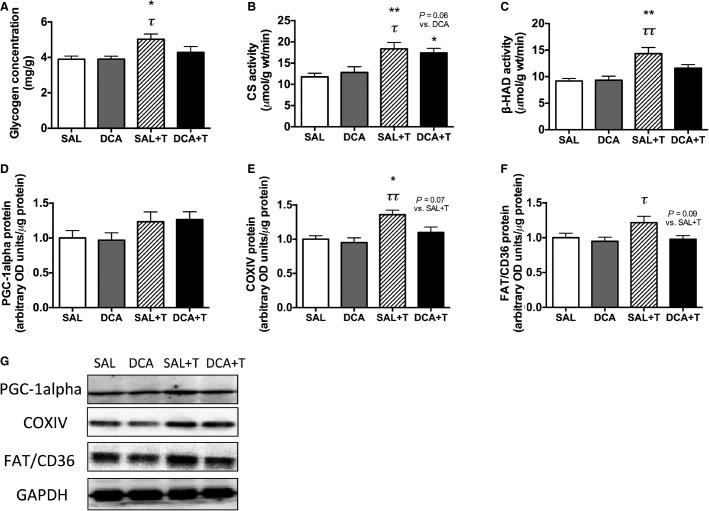
(A) Glycogen concentration, the activities of (B) CS and (C) *β*-HAD, and protein levels of (D) PGC-1alpha, (E) COXIV, and (F) FAT/CD36 in the white gastrocnemius muscle after 4 weeks of saline (SAL) or DCA (DCA) administration without training and high-intensity interval training with pre-exercise saline (SAL + T) or DCA (DCA + T) administration. Representative bands (G). Values are means ± SE (*N *=* *5–8). *P <* *0.05, **P <* *0.01 versus the SAL group. *τ P *<* *0.05, *ττ P *<* *0.01 versus the DCA group.

**Figure 4:**
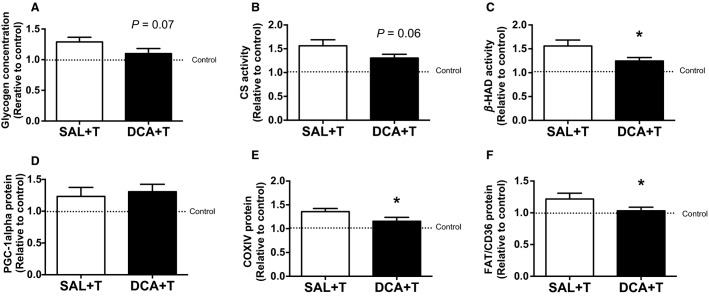
Training-induced increasing rates of (A) glycogen concentration, the activities of (B) CS and (C) *β*-HAD, and protein levels of (D) PGC-1alpha, (E) COXIV, and (F) FAT/CD36 in the white gastrocnemius muscle after 4 weeks. SAL + T and DCA + T groups were standardized by the mean of saline-treated control (SAL) and DCA-treated control (DCA) groups, respectively (Fig. 3A–F). Values are means ± SE (*N *=* *5–8). *P <* *0.05 versus the SAL + T group.

The data were expressed as the mean ± SE. Multigroup comparisons were performed by one-way analysis of variance (ANOVA), followed by the Tukey–Kramer post hoc test (blood lactate concentration in Table 3, Figs. 2, 3). Student’s *t*-test was used to examine the effect of DCA (muscle lactate and glycogen concentrations in Table 3, Figs. 1, 4). For all comparisons, statistical significance was defined as *P *<* *0.05.

**Table 3:** Lactate concentrations in blood and muscle and muscle glycogen concentration 10 min after saline or DCA administration and immediately after high-intensity interval exercise with pre-exercise saline or DCA administration

	Saline-treated sedentary	Saline-treated exercised	DCA-treated sedentary	DCA-treated exercised
Blood lactate (mmol/L)	1.46 ± 0.15	5.44 ± 0.40*τ	1.88 ± 0.38	4.03 ± 0.24*τ#
Muscle lactate (mmol/kg)	No data	20.84 ± 1.34	No data	16.61 ± 1.08#
Muscle glycogen (mg/g)	No data	2.29 ± 0.16	No data	2.62 ± 0.23

Values are means ± SE (*N *=* *6).

* *τ* and # Significant differences from the saline-treated sedentary, the DCA-treated sedentary, and the saline-treated exercised groups, respectively (*P *<* *0.05).

## Results

### Effects of pre-exercise DCA administration on metabolites (lactate and glycogen) and signal cascades after high-intensity interval exercise

We first checked whether pre-exercise DCA administration reduces lactate concentration in muscle and blood after high-intensity interval exercise. Blood lactate concentration increased after high-intensity interval exercise with or without DCA administration (*P *<* *0.05, Table 3). Immediately after 10 bouts of 1 min treadmill running at 40 m/min, lactate concentrations in blood (–25%, *P *<* *0.05) and the white gastrocnemius muscle (–20%, *P *<* *0.05) were lower in the DCA-treated exercised group than in the saline-treated exercised group (Table 3). These data confirmed that pre-exercise DCA administration decreased muscle and blood lactate accumulation after exercise, as observed previously (Hatta et al. 1991). Although lactate content was lower in the DCA group, the muscle glycogen concentration did not differ between the two groups after exercise (Table 3). We next measured the P-AMPK/T-AMPK, P-ACC/T-ACC, P-p38/T-p38, and P-CaMK/T-CaMK protein content ratios immediately after exercise (Fig. 1A–D). It is known that these signal cascades primarily regulate exercise-induced mitochondrial biogenesis in skeletal muscle (Egan and Zierath 2013), but these signals after exercise were not altered by DCA administration (Fig. 1A–D). However, the P-AMPK/T-AMPK and P-ACC/T-ACC ratios, which are indicators of AMPK signal activation, after exercise tended to be lower in the DCA group than in the saline group (Fig. 1A and B). Taken together, pre-exercise DCA administration decreased lactate concentrations in the body but did not affect the signal cascades involved in mitochondrial biogenesis after exercise.

### Effects of acute and chronic DCA administration on mitochondrial adaptations after high-intensity interval exercise

After we checked, by DCA administration, the reduction in lactate accumulation after exercise, we examined effects of acute and chronic DCA administration on exercise-induced mitochondrial adaptations.

#### Effects of acute DCA administration on mRNA expressions involved with mitochondrial biogenesis after exercise

To evaluate acute mitochondrial biogenesis after exercise, we measured mRNA expressions of PGC-1alpha and mitochondrial genes because these mRNA expressions, but not protein contents, drastically increase after acute exercise (Perry et al. 2010). CS, *β*-HAD, PGC-1alpha, and FAT/CD36 mRNA expressions were elevated 3 h after exercise (10 bouts of 1-min treadmill running at 40 m/min) in both saline- and DCA-injected mice (*P *<* *0.05, Fig. 2A–C and E) but COXIV mRNA expression increased only in saline-injected mice after exercise (Fig. 2D), indicating that reductions in lactate accumulation following DCA administration did not affect exercise-induced increases in mRNA expressions in most of the mitochondrial genes.

#### Effects of chronic DCA administration on exercise training–induced mitochondrial adaptations

There were no changes in body and muscle weights among the four groups after 4 weeks of exercise training, but fat weight was decreased significantly with 4 weeks of exercise training (Table 4, *P *<* *0.05). However, body, muscle, and fat weights were not significantly changed after the chronic DCA administration (Table 4). These results suggest that 4 weeks of chronic DCA administration did not affect basic physical parameters. We examined whether chronic DCA administration before exercise impaired training-induced increases in glycogen concentration, mitochondrial enzyme levels, and FAT/CD36, a main fatty acid transporter, protein content (Fig. 3A–G). There were no significant differences between the SAL and the DCA groups in glycogen concentration, mitochondrial and FAT/CD36 proteins (Fig. 3A–F). The exercise training increased glycogen concentrations (*P *<* *0.05), CS (*P *<* *0.01) and *β*-HAD (*P *<* *0.01) activity and COXIV (*P *<* *0.05) proteins in saline-administrated mice (Fig. 3A–C and E). However, in DCA-treated mice, glycogen concentration, *β*-HAD activity, and COXIV proteins were not changed after 4 weeks of training (Fig. 3A, C, and E). FAT/CD36 did not change after 4 weeks of training but the protein content in the SAL + T group tended to be higher than that in the DCA + T group (Fig. 3F, *P *=* *0.09). COXIV protein content also tended to be higher in the SAL + T group than the DCA + T group (Fig. 3E, *P *=* *0.07). PGC-1alpha did not significantly differ among all groups (Fig. 3D). Taken together, in DCA administered mice, general metabolic adaptations including increases in glycogen concentration, *β*-HAD activity, COXIV, and FAT/CD36 proteins were not induced after 4 weeks of training.

**Table 4:** Body, gastrocnemius muscle, and epididymal fat weights after 4 weeks of experimental period

	Saline-treated control	DCA-treated control	Saline-treated trained	DCA-treated trained
Body weight (g)	42.91 ± 1.35	42.31 ± 0.91	40.84 ± 0.99	42.04 ± 0.94
Gastrocnemius muscle weight (mg)	362.5 ± 9.28	372.4 ± 17.74	341.9 ± 18.67	378.9 ± 15.58
Epididymal fat weight (mg)	961.3 ± 164.0	841.9 ± 126.1	627.8 ± 41.13^*^	529.3 ± 30.75^*^τ
Epididymal fat weight (mg/g BW)	10.95 ± 1.53	9.90 ± 1.40	7.53 ± 0.55^*^	6.31 ± 0.38^*^τ

Values are means ± SE (*N* = 5–8).

Gastrocnemius muscle and epididymal fat were measured from both sides.

* and *τ* Significant differences from the saline-treated and the DCA-treated control groups, respectively (*P *<* *0.05).

In order to examine how much the parameters increased after 4 weeks of training in the DCA-treated and saline-treated groups, we calculated the training-induced increasing rate, which was demonstrated by the SAL + T and DCA + T groups that were standardized by the saline-treated control (SAL) and the DCA-treated control (DCA) groups, respectively (Fig. 4A–F). *β*-HAD activity and COXIV and FAT/CD36 proteins were significantly higher in the SAL + T group than the DCA + T group (*P *<* *0.05, Fig. 4C, E, and F). Glycogen concentration (*P *=* *0.07, Fig. 4A) and CS activity (*P *=* *0.06, Fig. 4B) also tended to be higher in the SAL + T group than the DCA + T group. DCA administration did not change PGC-1alpha protein content after 4 weeks of high-intensity interval training (Figs. 3D and 4D). These results suggested that DCA administration attenuated, in part, general muscle adaptations to exercise training such as increases in mitochondrial and fatty acid transporter protein levels and glycogen concentrations.

## Discussion

We examined whether reduced lactate accumulation following DCA administration alters mitochondrial adaptations after exercise. We first confirmed pre-exercise DCA administration reduced lactate accumulation in muscle and blood after high-intensity interval exercise without the changes in other signal cascades. We next examined effects of acute and chronic DCA administration on exercise-induced mitochondrial adaptations. Acute DCA administration-induced reductions in lactate accumulation during exercise did not affect the increases in the mRNA expression of mitochondrial genes except for COXIV after acute exercise. However, chronic DCA administration attenuated, in part, training-induced increasing rates of mitochondrial proteins (CS [*P *=* *0.06], *β*-HAD [*P *<* *0.05], and COXIV [*P *<* *0.05]), fatty acid transporter (FAT/CD36 protein [*P *=* *0.08]), and glycogen concentration (*P *=* *0.07) in the white muscle.

We first examined the effects of pre-exercise DCA administration on metabolite concentrations (lactate and glycogen) and signal cascades immediately after exercise. Pre-exercise DCA administration decreased muscle and blood lactate concentrations after 10 bouts of 1 min of treadmill running at 40 m/min (Table 3). These decreased lactate concentrations were consistent with previous reports using rodents (Schneider et al. 1981; Hatta et al. 1991; Durkot et al. 1995). Although glycogen concentrations after exercise were not significantly different between the saline- and the DCA-injected group, lactate accumulation was reduced when DCA was administrated. Similar with our study, previous studies demonstrated that lactate accumulation decreased in DCA infusions, but muscle glycogen concentrations after exercise were not significantly different between the DCA infusion and the control groups (Howlett et al. 1999; Parolin et al. 2000). Taken together, muscle and blood lactate accumulation during exercise was attenuated by pre-exercise DCA administration in this study.

There are several signal cascades of mitochondrial biogenesis, namely AMPK, CaMK, and p38 phosphorylation (Fig. 1). These signals acutely increase after endurance and high-intensity exercise and subsequently increase the transcription of PGC-1alpha and mitochondrial genes (Egan and Zierath 2013). We found that in the white gastrocnemius muscle immediately after exercise, the activation of these signals did not significantly differ between the saline and the DCA administration groups. Therefore, these data demonstrated that we could make the situation that only lactate concentrations are different without the changes in other signal cascades involved with mitochondrial biogenesis.

Several previous studies found that endurance and sprint exercises increase the mRNA expression of PGC-1alpha and mitochondrial genes (Hildebrandt et al. 2003; Wright et al. 2007; Perry et al. 2010; Little et al. 2011). In this study, 10 bouts of 1 min running (total of 19 min including rest) increased the mRNA levels of PGC-1alpha and mitochondrial genes such as COXIV, CS, *β*-HAD, and FAT/CD36 in the saline-administered group at 3 h after exercise. This is an important result indicating that total only 20 min of high-intensity interval exercise is sufficient to activate the transcription of mitochondrial and fatty acid transporter genes. As observed in the saline-injected group, DCA-injected mice exhibited increased mRNA levels of PGC-1alpha and mitochondrial genes at 3 h after exercise. However, COXIV mRNA did not increase after acute exercise in DCA-administrated group though it was not significantly different between the saline-treated exercise and the DCA-treated exercise groups. It is unclear why an exercise-induced increase in COXIV mRNA expression only diminished by DCA administration. However, Hashimoto et al. reported that COXIV mRNA expression was increased after lactate stimulation using the microarray analysis and real-time PCR (Hashimoto et al. 2007). Moreover, the fold change in the increase in COXIV by lactate was more significant than other genes involved in metabolism. Taken together, transcriptional activation in COXIV may be strongly affected by lactate than other mitochondrial proteins but the mechanisms are unclear.

After 4 weeks of high-intensity interval training, training-induced mitochondrial adaptations such as increases in mitochondrial enzyme activity (CS, *P *=* *0.06, and *β*-HAD, *P *<* *0.05) and protein (COXIV, *P *<* *0.05), were attenuated by DCA administration (Fig. 4). In addition, the increase in fatty acid transporter (FAT/CD36, *P *<* *0.05) and glycogen content (*P *=* *0.07), induced by exercise training concomitantly with mitochondrial adaptations, were also partially diminished by DCA administration (Fig. 4). These results raise the possibility that a reduction in lactate accumulation following pre-exercise DCA administration attenuates exercise-induced muscle mitochondrial biogenesis. Supporting this hypothesis, Hashimoto et al. found that incubation with a high concentration of lactate (10 and 20 mmol/L) for 6 h increased the mRNA expression of PGC-1alpha, a master regulator of mitochondrial biogenesis, and the DNA binding activity of transcriptional factors regulating mitochondrial genes using L6 cells (Hashimoto et al. 2007). Although not observed in a muscle study, lactate administration to mice in vivo increased PGC-1alpha mRNA expression in the liver (E et al. 2013). In agreement with these previous studies, unchanged COXIV protein content in DCA-treated group after 4 weeks of training would be attributed to weak transcriptional activation in COXIV in this study. In fact, COXIV mRNA and protein expressions were upregulated after 20 mmol/L lactate treatment (Hashimoto et al. 2007).

However, in the acute experiment, transcriptional activations in other genes involved with mitochondrial biogenesis were not different between the saline and the DCA administered groups (Fig. 2). This unexpected result in our study may be related to the use of high-intensity exercise in this study. For example, it has been reported that exercise-induced PGC-1alpha mRNA expression is exercise intensity dependent (Egan et al. 2010; Tobina et al. 2011). Therefore, it is considered that high-intensity exercise exerts stronger effects on mRNA upregulation after exercise compared with the difference in lactate concentrations using DCA administration. Although lactate concentrations were significantly lower in the DCA-treated exercise group than the saline-treated exercise group, muscle and blood lactate concentrations were 16.6 ± 1.08 mmol/kg and 4.0 ± 0.24 mmol/L, respectively, in the DCA group after the exercise (Table 3). These lactate concentrations could be enough for upregulating mitochondrial biogenesis at transcriptional level in mice. In addition, Latham et al. reported that lactate is a relatively weak inhibitor of histone deacetylase activity that promotes specific gene expression compared to other established inhibitors (Latham et al. 2012). Therefore, repetitive and cumulative lactate stimulation might be important and required to promote mitochondrial biogenesis in skeletal muscles because repeated transient mRNA bursts lead to increases in mitochondrial protein (Perry et al. 2010).

It is well known that red and white muscles have different lactate metabolic and transport capacity. Red muscle has more monocarboxylate transporter 1 (MCT1) protein, functions as the uptake of lactate to muscle, and white muscle has more MCT4 protein, functions as the release of lactate from muscle (Kitaoka et al. 2012). Moreover, mRNA expressions of metabolic genes were greatly increased in white muscles after high-intensity exercise (Hildebrandt et al. 2003). Therefore, there may be muscle fiber-type specific effects of lactate on training-induced mitochondrial biogenesis but we examined only white muscle in this study. We need to clarify the fiber-type specific effects of lactate on training-induced mitochondrial biogenesis in the future study.

## Limitation

We used pre-exercise DCA administration to reduce lactate accumulation during exercise. A limitation of this study is that we only measured lactate concentrations in muscle and blood. Although we believe that the decrease in lactate concentration was caused by DCA-induced PDH activation, lactate production and/or oxidation rates were unclear. Therefore, we should conclude that chronic training-induced mitochondrial adaptations were attenuated, in part, by decreased lactate “concentration” and/or “accumulation” following DCA administration.

Although we do not consider that DCA itself strongly affected our findings, we cannot completely exclude the effects of DCA itself because of some reasons. First, in the acute experiment, the DCA exposure time is different between the sedentary (10 min) and the exercised (29 min + 3 h post exercise) groups. Therefore, the DCA-treated exercised group was affected by DCA itself stronger than the DCA-treated sedentary group. However, in human skeletal muscle, 180 min infusion of DCA did not change gene expressions involved with substrate metabolism compared with the preinfusion state (Tisdale et al. 2011). Second, there are chronic effects of DCA administration. A previous study used chronic DCA treatment via drinking water demonstrated that DCA itself affects some physical parameters and liver glycogen concentrations (Kato-Weinstein et al. 1998). However, in the present study using i.p. injection, there is no significant difference between the saline- and the DCA-sedentary group in the chronic experiment (Table 4 and Fig. 3). Based on the data in previous studies and ours, DCA itself would not affect our findings though we cannot completely exclude the effects of DCA itself in this study.

## Conclusions

Pre-exercise DCA administration decreased lactate concentrations in muscle and blood without changes in other signal cascades during exercise. Acute DCA administration did not affect the upregulation of the mRNA expression of mitochondrial gene except for COXIV. Chronic DCA administration attenuated, in part, exercise-induced metabolic adaptations such as increases in mitochondrial enzyme activity (CS and *β*-HAD) and protein (COXIV), fatty acid transporter protein (FAT/CD36), and glycogen concentration. These results suggest that repeated lactate accumulation during exercise training may be associated with exercise training–induced mitochondrial adaptations.
